# Perceptual decision-making difficulty modulates feedforward effective connectivity to the dorsolateral prefrontal cortex

**DOI:** 10.3389/fnhum.2015.00498

**Published:** 2015-09-09

**Authors:** Bidhan Lamichhane, Mukesh Dhamala

**Affiliations:** ^1^Department of Physics and Astronomy, Georgia State UniversityAtlanta, GA, USA; ^2^Center for Behavioral Neuroscience, Neuroscience Institute, Georgia State UniversityAtlanta, GA, USA; ^3^Center for Nano-Optics, Georgia State UniversityAtlanta, GA, USA; ^4^Center for Diagnostics and Therapeutics, Georgia State UniversityAtlanta, GA, USA

**Keywords:** face-house categorization, dynamical causal modeling, FFA, PPA

## Abstract

Diverse cortical structures are known to coordinate activity as a network in relaying and processing of visual information to discriminate visual objects. However, how this discrimination is achieved is still largely unknown. To contribute to answering this question, we used face-house categorization tasks with three levels of noise in face and house images in functional magnetic resonance imaging (fMRI) experiments involving thirty-three participants. The behavioral performance error and response time (RT) were correlated with noise in face-house images. We then built dynamical causal models (DCM) of fMRI blood-oxygenation level dependent (BOLD) signals from the face and house category-specific regions in ventral temporal (VT) cortex, the fusiform face area (FFA) and parahippocampal place area (PPA), and the dorsolateral prefrontal cortex (dlPFC). We found a strong feed-forward intrinsic connectivity pattern from FFA and PPA to dlPFC. Importantly, the feed-forward connectivity to dlPFC was significantly modulated by the perception of both faces and houses. The dlPFC-BOLD activity, the connectivity from FFA and PPA to the dlPFC all increased with noise level. These results suggest that the FFA-PPA-dlPFC network plays an important role for relaying and integrating competing sensory information to arrive at perceptual decisions.

## Introduction

Humans are efficient in perceiving and discriminating the visual objects. How does the brain receive, relay, and integrate relevant sensory information to make such perception and discrimination known as perceptual decision? Specifically, what are the brain regions involved and how do these regions coordinate activity in perceptual decision-making processes? Previous studies showed that the brain areas on the ventral visual pathway process object category-specific visual information (Kanwisher et al., 1997; Haxby et al., 2000, 2001, 2002; Engell and Mccarthy, 2010). However, visual information processing in these early visual areas was found insufficient in discrimination of visual objects (Rossion et al., 2003; Avidan et al., 2005; Avidan and Behrmann, 2009). In spite of the abundant research in the field (Wilson et al., 1993; Haxby et al., 2000, 2002; Ishai et al., 2005; Fairhall and Ishai, 2007; Heekeren et al., 2008; Ishai, 2008), we do not exactly know where and how visual information is processed in the brain to arrive at a difficult perceptual decision. In this study, we used face-house categorization tasks with three levels of noise in face-house images in functional magnetic resonance imaging (fMRI) experiments to answer these questions.

The encoding of relevant sensory information is one of the main steps of the brain processes in the cognitive chain leading to perceptual decisions. Experiments on both humans and non-human primates have demonstrated that the first stage of perceptual decision-making involves lower order regions receiving and representing sensory information (Newsome and Paré, 1988; Britten et al., 1992; Salzman et al., 1992; Romo et al., 1998; Hernández et al., 2000; Binder et al., 2004). For example, perception of faces showed stronger response in the fusiform face area (FFA; Kanwisher et al., 1997) and that of house in the parahippocampal place area (PPA; Aguirre et al., 1998; Epstein and Kanwisher, 1998; Haxby et al., 2001; Vuilleumier et al., 2001) and interaction between these regions is important in perception of face and house (Stephan et al., 2008). However, relatively recent studies in the field have shown that the representation of visual information in these areas, also called core system, is not sufficient (Marotta, 2001; Avidan et al., 2005; Schiltz et al., 2006; Avidan and Behrmann, 2009), and further processing of visual information in the higher order cortical area, also called the extended system, is crucial to discriminate visual objects (Fairhall and Ishai, 2007; Heekeren et al., 2008; Avidan and Behrmann, 2009).

The frontal cortex activity, especially activity in the dorsolateral prefrontal cortex (dlPFC), was reported in semantic analysis (Gabrieli et al., 1998), disambiguation (Carlson et al., 2006), and temporal processing (Smith et al., 2003). The dlPFC was also found to be involved in social decision-making (Sanfey et al., 2003; Knoch et al., 2006; Lamichhane et al., 2014) and cognitive control (Miller and Cohen, 2001). The dlPFC has been understood to accumulate relayed sensory information to form a decision (Bar et al., 2001). In a previous study, similar to ours, the core system was found to be functionally organized in a hierarchical, feed-forward architecture, in which the core exerted a strong causal influence on the extended system in frontal cortex (Fairhall and Ishai, 2007).

However, the area-specific activity alone, such as activity in the ventral cortex and dlPFC, has been suspected not to be sufficient for the perception of faces (Simon et al., 2011). Thus, the neural underpinning underling ability of visual perception remain unclear and understanding of how these regions in core and extended system coordinate activity in relaying and integrating competing sensory information to arrive at perceptual decisions is very important. Here, we aimed to map out the neural mechanisms for perceptual decision-making processes by examining categorization-task specific brain activations, brain connectivity and their modulations by decision-making task difficulty. We included pictures of faces and houses as the stimuli and hypothesized that there would be a significant connectivity from FFA and PPA to the dlPFC during face-house categorization. We also added three levels of noise in our stimuli and predicted that there would be an increase in connectivity within category-specific brain areas in the ventral temporal (VT) region (the FFA and the PPA) and feedforward connectivity between these regions and the extended system (the dlPFC) by face-house categorization difficulty. The rationale for this prediction is based on the notion that as noise in face-house stimuli increases, the neural representation of category specific information in FFA and PPA decreases (Heekeren et al., 2004). As the result of decrease in category specific information in these regions with noise, the sparse sensory information has to be gathered and evaluated and potentially increasing decision-related brain activity in decision-making processes (Gabrieli et al., 1998; Bar et al., 2001; Carlson et al., 2006; Hernández et al., 2010).

## Materials and Methods

### Participants

Thirty-three human participants (17 females; mean age 27.54 ± 4.67 years) participated in this study. All participants had normal or corrected to normal vision and reported normal neurological history. Participants provided written signed informed consent forms and were compensated for their participation in the experiments. Institutional Review Board (IRB) for Joint Georgia State University and Georgia Institute of Technology Center for Advanced Brain Imaging, Atlanta, GA, USA approved this study.

### Stimuli

**Figure 1:**
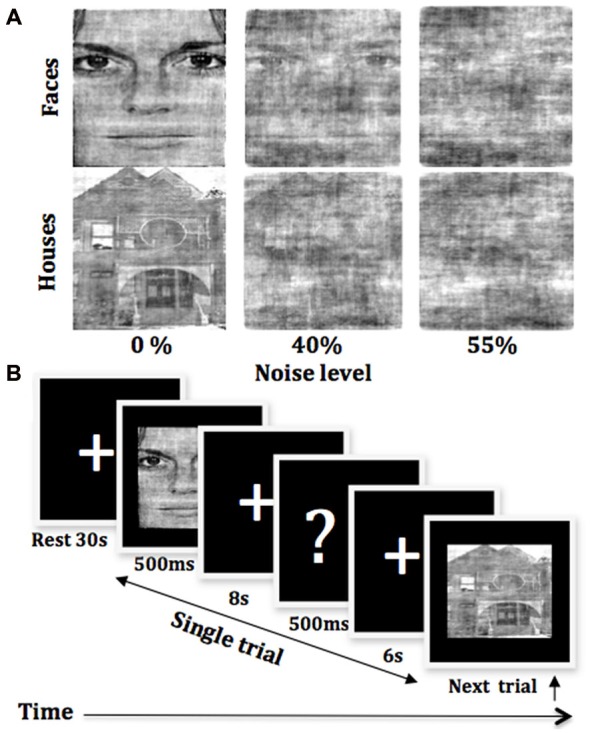
**Experimental paradigm. (A)** Sample images at three noise levels for sets of both face and house stimuli. **(B)** Task paradigm during a functional run, starting from the initial 30 s rest followed by a task trial that included 500 ms-stimulus presentation, 8 s of decision time, and 500 ms-display of a question mark, requiring participants to indicate their decision within the next 6 s.

We used a total of 14 neutral images of faces and 14 images of houses as stimuli. All the presented pictures were downloaded from F.A.C.E. Training–an interactive training by Paul Ekman.^1^ All the images were equalized for luminance and contrast by converting them to gray scale and were cropped to make equal size. Furthermore, both face- and house- images were degraded by manipulating images and adding noise (Rainer and Miller, 2000). Image pixel phase randomization and addition of Gaussian noise enabled us to make visual image stimuli noisy. Stimuli consisted of three different noise levels: 0, 40 and 55%, for both sets of images (Figure 1A). The stimulus software Presentation^2^ was used to display stimuli and to control task trial sequences.

### Task and Behavioral Paradigms

The experimental task was divided into two separate sessions: the first session involved acquiring behavioral data outside the MRI scanner and the second session was inside the scanner, where we acquired both fMRI and behavioral data. In both cases, participants were asked to decide whether the presented gray scale images were faces or houses. They indicated their decisions by keyboard or button presses on a response box. Prior to the experimental tasks, participants were briefly explained about the study and the task. Some sample stimuli were shown and the participants were asked to make decisions about the presented stimuli, allowing them to be familiar with the task.

### Outside the fMRI Scanner

This behavioral study consisted of a single run. There were three noise conditions and each condition was repeated 60 times (30 times each for faces for houses) in a random order, generating 180 trials in total. Participants were asked to indicate their decisions as quickly and as accurately as possible by the right and left mouse clicks (right for house stimuli and left for face stimuli). They were instructed to press the space bar in the computer keyboard to proceed to the next trial. The type of stimuli, the stimulus presented times, and the response times (RTs) to that stimuli were all recorded.

### Inside the fMRI Scanner

Participants performed face-house categorization tasks in three functional runs, each 614 s long. The number of trials for each noise condition was 36 (18 faces and 18 houses), and the total trials were 108 for all three conditions in each run. Stimuli were presented in a random order as in an event-related design within each run. There were rest periods of 30 s at the beginning and of 35 s at the end of each run. Participants were instructed to focus on the central crossbar on the screen during experimental run. They were asked to perceive the presented stimuli, to wait for the display of a question mark on the screen and then to indicate their choice by pressing a response key on a button-box by using either the index or the middle finger of their right hand. Each picture was presented for 500 ms, followed by an 8 s-long display of the fixation cross, then a briefly presented question mark for 500 ms at the end of this 8 s’ interval. The next 6 s time was allowed for participants to report their decisions by responding on a button box. Trials in which participants were failed to respond were discarded from the final analysis. Figure 1B shows a schematic representation of the behavioral paradigm used in the experiment.

### Data Acquisition and Analysis

#### Behavioral Data

A participant’s RT, the time between the onset of a stimulus and the button press in each trial was recorded for the tasks performed outside the scanner. Participants were required to do button presses only to indicate their decisions inside the scanner. Participants’ behavioral performance, both outside and inside the scanner, was analyzed by using Matlab. Trial by trial RTs of each participant from outside-scanner button presses were separated and averaged across noise conditions. No RT calculation was done for the recorded behavioral data inside the scanner as participants were instructed to wait until the question mark was displayed to indicate their decisions. *T*-tests were used to assess the significance levels of performance accuracy and RT across noise levels in face-house stimuli. The behavioral responses (RT and performance accuracy) were collected from 32 participants.

#### Functional Magnetic Resonance Imaging (fMRI) Data

The whole-brain MR imaging was done on a 3-Tesla Siemens scanner available at Georgia State University and Georgia Institute of Technology Center for Advanced Brain Imaging (CABI), Atlanta, Georgia. High-resolution anatomical images were acquired for anatomical references using an MPRAGE sequence (with TR = 2250 ms, TE = 4.18 ms, Flip angle = 90°, inversion time = 900 ms, voxel size = 1 × 1 × 1 mm^3^). Three functional runs each of 307 scans with the measurement of the T2*-weighted blood-oxygenation level dependent (BOLD) effect, were acquired with a gradient echo-planar imaging protocol and these parameters: echo time (TE) = 30 ms, repetition time (TR) = 2000 ms, flip angle = 90°, voxel size = 3 × 3 × 3 mm^3^, field of view = 204 mm × 204 mm, matrix size = 68 × 68 and 37 axial slices each of 3 mm thickness.

FMRI data were analyzed by using Statistical Parametric Mapping (SPM8, Wellcome Trust Center, London).^3^ The analysis steps included slice timing correction, motion correction, co-registration to individual anatomical image, normalization to Montreal Neurological Institute (MNI) template (Friston et al., 1994). Spatial smoothing of the normalized image was done with an 8 mm isotropic Gaussian kernel. A random-effects model-based univariate statistical analysis was performed in two level procedures. At the first level, a separate general linear model (GLM) was specified according to the task sequences and behavioral responses for each participant. Only correct trials for each of the three noise-levels (0, 40 and 55%), rest and six motion parameters were included in GLM analysis. Here, six motion parameters were entered as nuisance covariates and were regressed out of the data. The individual contrast images of all participants from the first level analysis were then submitted into a second level analysis for a separate one-sample *t*-test (for details of the contrasts used in first level and corresponding second level, please see section: Brain Activity and Effective Connectivity Analysis and Table 1). The resulting summary statistical maps were then threshold and overlaid on high-resolution structural images in MNI orientation. For display purposes, the functional images were overlayed on the MNI template available in MRIcro.^4^

**Table 1:** **Brain activations of face-and-house perception**.

		MNI coordinates				
Contrast	Brain area	*x*	*y*	*z*	Cluster size	*Z*-score
Face > House*	Fusiform face area (FFA)	42	−49	−17 (R)	31	4.33
House > Face*	Parahippocampal place area (PPA)	−27	−46	−8 (L)	27	5.64
All pictures > Rest**	Inferior parietal lobe (IPL)	−27	−58	46 (L)	12	5.69
	Pre-supplementary motor area (Pre-SMA)	−3	14	49 (L)	49	6.64
	Dorsolateral prefrontal cortex (dlPFC)	42	8	25 (R)	32	6.35
	Insula	33	26	7 (R)	38	6.29
		−30	26	1 (L)	24	6.15
	Venteral temporal cortex (VT)	30	−46	−14 (R)	489	7.62
		−27	−55	−11 (L)	383	9.91
	Occipital cortex	15	−85	−8 (R)	489	9.42
		−12	−100	−4 (L)	383	7.13
	Posterior cingulate cortex (PCC)	12	−70	13 (R)	41	6.06

R, Right, L, left. Family-wise error corrected (FWC) at *p << 0.05 and ***p << 0.001.

#### Brain Activity and Effective Connectivity Analysis

We examined the brain activity of hypothesized regions of interest (ROIs) in our experimental condition (i.e., face-house discrimination task at different noise-levels). We defined the ROIs from the group level activation results. To localize FFA activation in-group level, we used face > house contrast. Similarly to localize PPA, we contrasted house with face (house > face). The peak-activity location of the dlPFC was chosen using face + house > rest contrast. The ROIs analysis were performed using a spherical region of 6 mm radius centered at the maxima peak activity voxel of group level result using MarsBaR (Brett et al., 2002). The beta parameters (also called contrast values) were extracted for each experimental condition that was defined in design matrix for each subject. The beta parameters of condition of interest were then averaged over the subjects. Finally, statistical tests (a paired *t*-test following a repeated ANOVA) were performed to determine whether there was a statistically significant difference in contrast values between the conditions of interests.

The effective connectivity established by our experimental conditions between ROIs were examined using dynamical causal modeling (Friston et al., 2003; Marreiros et al., 2008; Stephan et al., 2009) implemented in SPM8 (DCM10). For this purpose, we used group level peak activity coordinates as a reference to find the local maxima from the first level brain map. Then we extracted the eigenvariate by defining a sphere of radius 6 mm for the contrast of interest adjusted for the equivalent F-contrast. While extracting eigenvariate, the center of each ROI was positioned on the most significant voxel in the cluster nearest to the peak cluster coordinate obtained from group analysis and activated at a significant level (*p* < 0.1, uncorrected) and lie within twice the width of the Gaussian smoothing kernel used while smoothing the data. The details of modal specification and comparison procedure were included below.

## Results

### Behavioral Response

**Figure 2:**
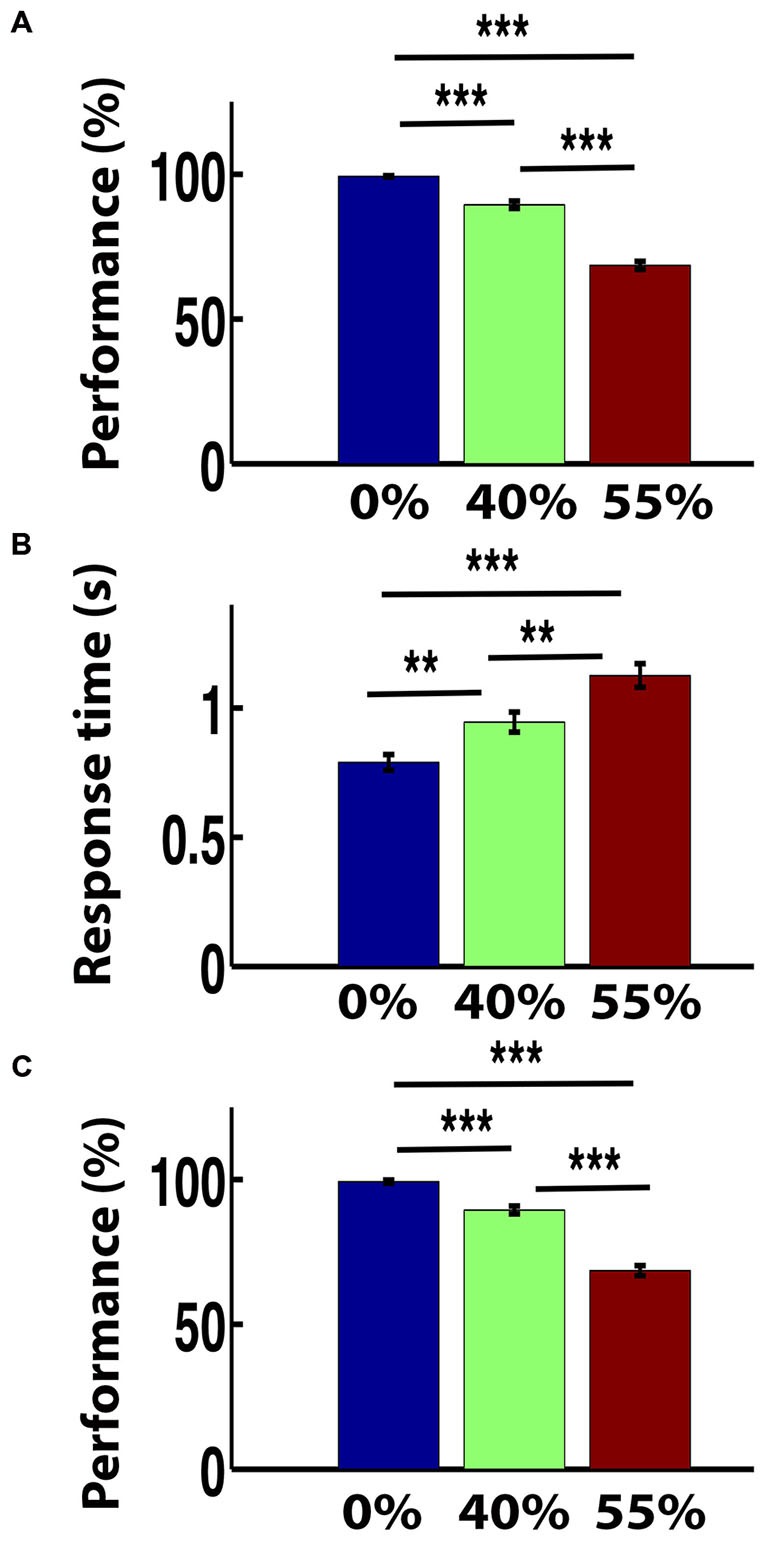
**Behavior response.** The bar plots of **(A)** mean performance (%) outside the scanner, **(B)** response time (RT) outside the functional magnetic resonance imaging (fMRI) scanner and **(C)** mean performance (%) inside the fMRI scanner for three noise-levels. Error bars show standard error of the mean. **p < 0.01 and ***p < 0.001.

The mean performance (i.e., the group level accuracy) for images with 0% noise-level was very high. The accuracy rate for 0% noise was 99.26% for outside scanner and that for inside the scanner was 97.89%. The performance level decreased for 40% noise-level and the rates were 89.48 and 87.01% for outside and inside the scanner respectively. The rates further decreased to 68.52 and 65.07% for outside and inside the scanner respectively when the noise level increased to 55%. A repeated ANOVA on the performance and RT revealed the significant effect of noise (of task difficulty) on behavioral performance (*F*_(2,62)_ = 265.02, *p* = 0.000 (outside the scanner) and *F*_(2,62)_ = 186.34, *p* = 0.000 (inside the scanner) and on RT *F*_(2,62)_ = 76.2, *p* = 0.000). Following significant ANOVA, a *post hoc* paired *t*-test was preformed. The decrease in performance with three noise levels was statistically significant both inside and outside the scanner (paired *t*-test, all *p* < 0.001; Figures 2A,C). Similarly RTs significantly increased with noise level (paired *t*-test, all *p* < 0.01). The mean RT for clear images (0% noise) was 0.79 s and that for 40% noisy-images was 0.94 s. The RT further increased to 1.13 s for 55% noise level (Figure 2B).

### Brain Activations

**Figure 3:**
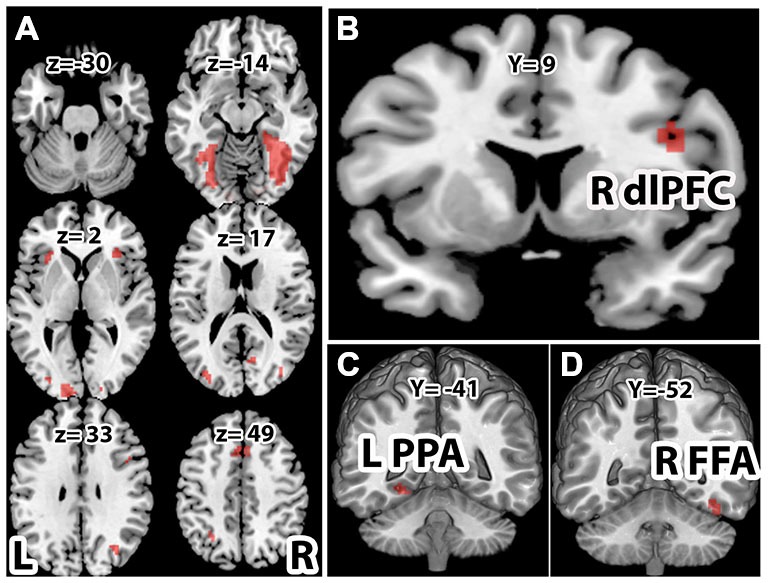
**Brain activations.** Activations associated with **(A)** face and house stimuli > rest (*p* < 0.001). **(B)** Right dlPFC for face and house > rest (*p* < 0.001). **(C)** Left parahippocampal place area (PPA) for house > face (*p* < 0.05), and **(D)** right fusiform face area (FFA) for face>house (*p* < 0.05). All activations are familywise error corrected (FWC). For the display purpose, the functional images were overlayed on the Montreal Neurological Institute (MNI) template available in MRIcro and the coordinates of brain activation were shown in the Table 1.

**Figure 4:**
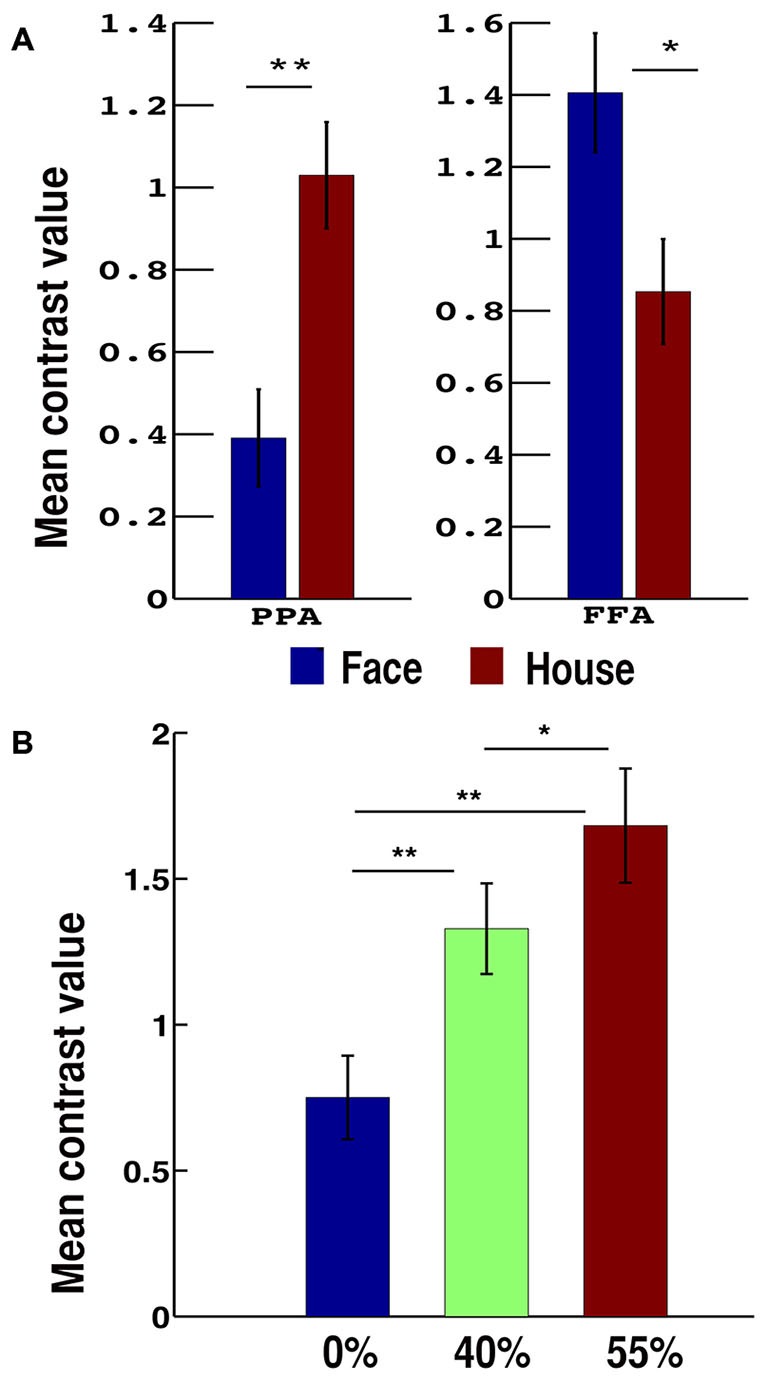
**Bar plots of mean contrast values.** **(A)** For face and house presented conditions in PPA and FFA. **(B)** According to the noise-level in dorsolateral prefrontal cortex (dlPFC). Error bars show standard error of the mean. *p < 0.05 and **p < 0.01.

With the face-house decision vs. rest contrast, we observed significant brain activations in the occipital, lateral occipital cortex (LOC), FFA and PPA in the VT cortex (VT), inferior parietal lobe (IPL), dlPFC, insular cortex (INS), pre-supplementary motor cortex in middle frontal cortex (Pre-SMA; Figures 3A,B). To localize the category specific brain regions in VT, we further contrasted face vs. house and house vs. face conditions (Table 1). The face vs. house contrast showed a stronger response in the FFA (Figure 3D). Similarly, the house vs. face contrast activated PPA more (Figure 3C). The ROI analysis showed higher BOLD responses for face in FFA (a repeated ANOVA, (*F*_(1,32)_ = 17.34, *p* = 0.0002 and paired *t*-test, *p* < 0.05) and that of house in PPA (a repeated ANOVA, (*F*_(1,32)_ = 48.55, *p* = 0.000 and paired *t*-test, *p* < 0.01; Figure 4A). Similarly, a repeated ANOVA was performed to find the effect of noise on dlPFC activity (*F*_(2,64)_ = 17.27, *p* = 0.000) and BOLD activity increased with noise level (paired *t*-test, all *p* < 0.05; Figure 4B).

### Dynamic Causal Modeling (DCM) Results

**Figure 5:**
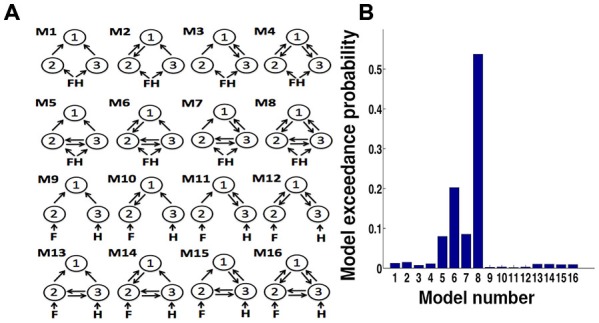
**(A)** Dynamical causal models (DCM) model specifications. Model number 1 is a basic model that included the minimal number of connections between dlPFC (1) with FFA (2) and PPA (3). The endogenous connectivity of this “minimal” model was then modified by systematically adding connections (from model 2 to 4). Models 5–8 were construct model from 1 to 4 by adding bidirectional connections between FFA and PPA. In these models (1–8), all face-house images were the inputs to both FFA and PPA. We further used face image input to FFA and house input to PPA and expanded our model space to 16. **(B)** Bar plots of exceedance probability for 16 models.

Choosing a functional architecture of a network of hypothesized ROIs is very important in dynamical causal models (DCM) analysis. To determine which model most likely generated the data (for example; whether the data is best explained by bottom-up, or in combination with top– down, or considering the presence/absence of connections), we considered various endogenous DCM (Kahan and Foltynie, 2013). First, we defined eight models for the network consisting of the dlPFC (region 1), FFA (region 2) and PPA (region 3; Figure 5A). The “minimal” model (model 1) was systematically modified by adding connections to build other models (model 2 to model 8). In these eight models, both face and house trials were used as input on both FFA and PPA. However, the other important factor is deciding which input to be considered as a driving input (for example, what input to be provided in a particular node?). Thus, we considered additional eight models (model 9 to16) in which face stimuli were considered to be the input to FFA and house stimuli to PPA. We designated bidirectional connections between FFA and PPA and inputs to FFA and PPA (not to dlPFC) based on our hypothesis and evidence provided by previous studies in the field (Kanwisher et al., 1997; Aguirre et al., 1998; Epstein and Kanwisher, 1998; Haxby et al., 2001; Marotta, 2001; Vuilleumier et al., 2001; Avidan et al., 2005; Schiltz et al., 2006; Fairhall and Ishai, 2007; Heekeren et al., 2008; Stephan et al., 2008; Avidan and Behrmann, 2009). The random effects Bayesian model selection procedure (BMS) was then used to select the optimal model at the group level (Stephan et al., 2009). Out of 16 plausible intrinsic models, between FFA, PPA and dlPFC, the model 8 consisting of bidirectional connections between all the ROIs came out to be the optimal model with exceedance probability (xp) = 0.54 (Figure 5B). So for further DCM analysis, we kept matrix A, the matrix of intrinsic connections, fully connected between ROIs across all models and both face and house trials were used as input on both FFA and PPA.

In order to quantify effective connectivity, we considered four types of models by allowing varying connection to be modulated by task. In all models contextual modulation was allowed to the connection between FFA and PPA (i.e., both FFA to PPA and PPA to FFA connection). The models were then systematically varied allowing further modulation in connection between brain areas. The first model was constructed by allowing feedforward connection (FFA and PPA to dlPFC) to be modulated by task in addition to FFA to PPA and PPA to FFA connections. The second model was then constructed by further modulating dlPFC to FFA connection in the first model. The third model consisted of an additional dlPFC to PPA modulation on the first model. Finally, the fourth mode was designed by allowing all the connections to be modulated by the task.

**Figure 6:**
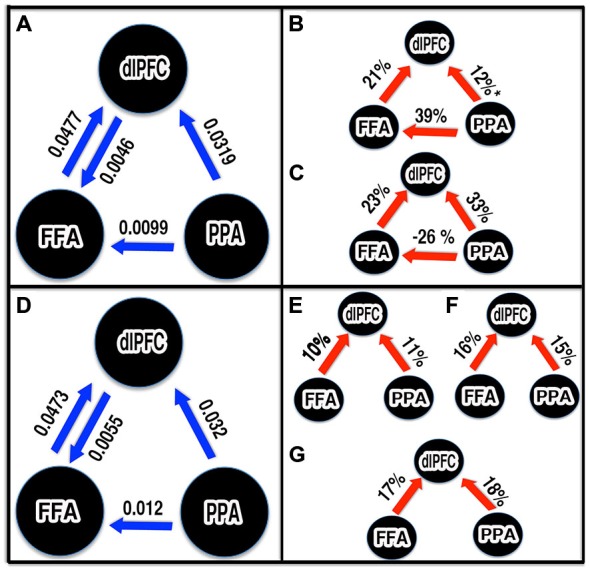
**Schematic representations of significant connections and parameter estimates from Bayesian model averaging (BMA).** Unless stated otherwise, all connections shown were statistically significant (*p* < 0.05). The blue arrows: significant intrinsic connectivity between nodes. The number next to the blue arrow represents the connection strength. The red arrow: significant increased in effective connectivity. The number next to red arrow represents percentage increased in connectivity due to task. **(A,D)** Intrinsic connectivity pattern between FFA, PPA and dlPFC established by first and second sets of DCM. **(B,C)** Modulation during face and house viewing condition respectively. **(E–G)** Modulation by 0%, 40% and 55% noise stimuli respectively. *p = 0.06, marginally significant.

We performed two sets of DCM analysis. The first set of DCM models was object-category specific: face and houses, regardless of noise, were used modulatory inputs to the models described above. Then, the random effects Bayesian model averaging (BMA) procedure (BMA.rfx) was used to compute resultant patterns of connection strengths (intrinsic and modulatory) established by the perception of faces and houses. The intrinsic connections between nodes (except dlPFC to PPA and FFA to PPA) were found significant (*t*-test, *p* < 0.05; Figure 6A). Next, we investigated whether the connections were modulated by picture category presented. In the face conditions, the connectivities from FFA to dlPFC and PPA to FFA were significantly modulated (*t*-test < 0.05) and PPA to dlPFC was also found marginally significant (*p* = 0.06; Figure 6B). In house conditions, all connections were significantly modulated (*t*-test < 0.05) except dlPFC to FFA and PPA connections (Figure 6C). Additionally, the face viewing enhanced the FFA to dlPFC connectivity much higher (by 21%) compared PPA to dlPFC (by 12%). Similarly, the house condition boosted connectivity from PPA to dlPFC by 33% where as the FFA to dlPFC increased by 23%. The connectivity from PPA to FFA was found increased by face but decreased by house condition by 36 and 26% respectively.

In the second set of DCM models, three noise conditions (0, 40 and 55%), independent of face and house, were allowed to modulate the connections. In this set too, we considered four models as in first set and the modulation matrix (B-matrix) was defined per noise condition as in first set. After BMA, the intrinsic connections from FFA and PPA to dlPFC, PPA to FFA, and dlPFC to FFA were found significant as in the first set (Figure 6D). Our modulation parameter analysis further revealed that all feedforward connections from FFA and PPA to dlPFC were significantly (*t*-test, *p* < 0.05) modulated my noise (Figures 6E–G). The FFA to dlPFC connectivity strengths were increased by 10, 16, and 17% for images of noise level 0, 40 and 55% respectively. Similarly, PPA to dlPFC connectivity was enhanced by 11, 15 and 18% respectively in 0, 40 and 55% noise level.

## Discussion

We investigated the brain activity, effective connectivity and their modulations by task in visual stimuli-directed perceptual decision-making. Consistent with previous findings (Haxby et al., 2000, 2001, 2002; Leveroni et al., 2000; Ishai et al., 2005; Ishai, 2008), we identified two category-responsive regions FFA and PPA in VT area of the brain and the prefrontal region (dlPFC). We computed resultant connection (intrinsic and modulatory) strengths established by our task. In one set of models, we allowed face and house conditions to modulate various connections between nodes in an optimal neural architecture and in the other set according to noise level. Using BMA, we consistently observed significant feedforward connectivity from category-responsive regions to the dlPFC during perceptual decision of faces and houses in both sets of DCM.

The observed significant intrinsic connectivity, also known as average connectivity established by task, from category-responsive brain regions to more anterior regions of the brain, the dlPFC, in particular, is consistent with the proposal that ventral visual system is the pathway for relaying and processing sensory information of visual objects (Kanwisher et al., 1997; Aguirre et al., 1998; Epstein and Kanwisher, 1998; Haxby et al., 2001; Vuilleumier et al., 2001).These results are also consistent with the function of the visual system that it may not be involved in a higher order perceptual analysis (Ploran et al., 2007) but may provide a causal input to the extended system (Mechelli et al., 2004; Fairhall and Ishai, 2007; Kveraga et al., 2007) and the relayed sensory information is further processed by downstream processing areas to produce visual perception (Rossion et al., 2003; Avidan et al., 2005).

In addition to the significant feed-forward intrinsic connectivity from FFA and PPA to dlPFC, we also observed significant feed-back intrinsic connectivity from dlPFC to FFA which underscores the importance of feedback mechanism in processing of visual information (Haxby et al., 2000). This top- down (or feedback) connectivity might regulate the bottom-up process of visual processing (Mechelli et al., 2004; Summerfield et al., 2006). These findings show that the involvement of both bottom-up and top-down processes are necessary for successfully evaluating visual stimuli consistent with previous studies (Haxby et al., 2000; Mechelli et al., 2004; Summerfield et al., 2006; Ishai, 2008; Li et al., 2009).

The feedforward connectivity from FFA and PPA to dlPFC was found modulated by perception of both face and house. This evidence as well as the observed the high BOLD response in FFA and PPA for non-prefered category (Figure 4A) favors the hypothesis that the FFA and PPA not only each process its preferred category but also represents the other form of visual objects (for example, non-preferred category) and their physical properties (Ishai et al., 1999; Haxby et al., 2000, 2001; Freedman et al., 2002; Kiani et al., 2007; Li et al., 2009). Furthermore, the stronger the modulation of FFA to dlPFC connectivity (by 21%, increased compared with intrinsic connectivity) by faces compared to PPA to dlPFC (12%), the higher BOLD response in FFA for face, and the stronger the modulation of PPA to dlPFC connectivity (by 33%) by house compared to FFA to dlPFC (23%), the higher BOLD response in PPA for houses, which supports area-specific dominant roles on face and house processing as purposed by many previous studies (Kanwisher et al., 1997; Aguirre et al., 1998; Epstein and Kanwisher, 1998; Haxby et al., 2001; Vuilleumier et al., 2001). Our DCM results also favored the neural interaction between FFA and PPA. This supports the importance of interactions between these regions in visual processing (Rossion et al., 2003; Sorger et al., 2007; Stephan et al., 2008).

We have measured the decision-making difficulty behaviorally both in terms of performance accuracy and RT. The result showed that noise added to face or house images made perceptual categorization decisions difficult. In brain level, the modulation of feedforward connectivity to dlPFC and brain activity in dlPFC by task difficulty was consistent with the notion that the brain requires more effort to accumulate sensory information together from ambiguous sensory information before a decision about stimulus category can be formed (Gabrieli et al., 1998; de Lafuente and Romo, 2005; Carlson et al., 2006; Hernández et al., 2010). Here, the function of the dlPCF also fits with its role in disambiguation (Carlson et al., 2006), in semantic analysis involved in recognition (Gabrieli et al., 1998) and in decision making (Miller and Cohen, 2001; Pasupathy and Miller, 2005; Weissman et al., 2008). The greater response to the noisy but recognized stimuli (Bar et al., 2001) in dlPFC further supports its role in evaluation of sensory information (Hernández et al., 2010).

We focused our analysis on category-responsive regions in VT area of the brain (Kanwisher et al., 1997; Aguirre et al., 1998; Epstein and Kanwisher, 1998; Haxby et al., 2001; Vuilleumier et al., 2001) and the dlPFC, the decision making hub (Gabrieli et al., 1998; Bar et al., 2001; Miller and Cohen, 2001; Rossion et al., 2003; Sanfey et al., 2003; Smith et al., 2003; Mechelli et al., 2004; Avidan et al., 2005; Carlson et al., 2006; Knoch et al., 2006; Fairhall and Ishai, 2007; Kveraga et al., 2007; Ploran et al., 2007). Other brain regions such as Pre-SMA, bilateral IPL and bilateral the INS were also activated by the task. However, we excluded these regions in DCM analysis as these regions are known for supporting cognitive processes such as attention, working memory (Lau et al., 2004; Olson and Berryhill, 2009). The peak activation coordinates for pre-SMA obtained in our study are close to the peak activity locations reported in previous studies and was found associated with the attention (Pessoa et al., 2003; Heekeren et al., 2004, 2008). The insular activation is known to related with the subjective experience of emotional states and feelings (Sterzer and Kleinschmidt, 2010). Similarly, IPL is known to be involved in visual short-term memory (Marois et al., 2004; Marois and Ivanoff, 2005; Olson and Berryhill, 2009). However, we exclude IPL from our DCM analysis mainly because: IPL is not a part of ventral processing stream (Ungerleider et al., 1998; Kravitz et al., 2013) for mediating the visual recognition of objects (“what” an object is) and the choice of a fewer nodes also worked in our favor for the DCM analysis since a large number of nodes in DCM analysis can be computationally expensive and at times, problematic (Stephan et al., 2010).

Summarizing, we showed how the dynamics of distinct cortical areas contributes to the processing of visual-sensation that leads to perceptual decisions. In relation to our task, evidence supports us to argue that the FFA-PPA-dlPFC network represents a minimal brain circuitry necessary for relaying and integrating competing sensory information, and has a role in perceptual decision-making. Future studies using this type of experiment in multisensory domains can lead to uncovering brain functional architectures necessary for more complex perceptual decision-making processes in the brain.

## Conflict of Interest Statement

The authors declare that the research was conducted in the absence of any commercial or financial relationships that could be construed as a potential conflict of interest.

## Footnotes

^1^https://www.paulekman.com/product/pictures-of-facial-affect-pofa/

^2^http://www.neurobs.com

^3^http://www.fil.ion.ucl.ac.uk/spm

^4^http://www.mccauslandcenter.sc.edu/CRNL
